# Is there a role for sacral neuromodulation in patients with neurogenic lower urinary tract dysfunction?

**DOI:** 10.1590/S1677-5538.IBJU.2020.99.10

**Published:** 2020-09-02

**Authors:** Marcio Augusto Averbeck, Jorge Moreno-Palacios, Alejandro Aparicio

**Affiliations:** 1 Unidade de Videourodinâmica Moinhos de Vento Hospital Porto AlegreRS Brasil Coordenador de Neurourologia, Unidade de Videourodinâmica, Moinhos de Vento Hospital, Porto Alegre, RS, Brasil;; 2 Unidad de Alta Especialidad Centro Médico Nacional Siglo XXI, IMSS México Servicio de Urologia, Unidad de Alta Especialidad Centro Médico Nacional Siglo XXI, IMSS, México, MX;; 3 Clinica del Country Bogota Colombia Clinica del Country, Bogota, Colombia

**Keywords:** Urinary Tract, Lower Urinary Tract Symptoms, Transcutaneous Electric Nerve Stimulation

## Abstract

**Purpose:**

To review current literature regarding sacral neuromodulation (SNM) for neurogenic lower urinary tract dysfunction (NLUTD) focused on indications, barriers and latest technological developments.

**Material and Methods:**

A PubMed database search was performed in April 2020, focusing on SNM and various neuro-urological conditions.

**Results:**

SNM has been increasingly indicated for lower urinary tract dysfunction (LUTD) in neuro-urological patients. Most studies are cases series with several methodological limitations and limited follow-up, lacking standardized definition for SNM clinical success. Most series focused on neurogenic overactive bladder in spinal cord injured (incomplete lesions) and multiple sclerosis patients. Barriers for applying this therapy in neurogenic LUTD were mainly related to magnetic resonance imaging incompatibility, size of the implantable pulse generator (IPG), and battery depletion. Newer technological advances have been made to address these limitations and will be widely available in the near future.

**Conclusions:**

SNM seems a promising therapy for neurogenic LUTD in carefully selected patients with incomplete lesions. Further studies are still needed to define which subgroups of neurological patients benefit the most from this minimally invasive technique.

## INTRODUCTION

Sacral neuromodulation (SNM) is an established third-line treatment for idiopathic lower urinary tract dysfunctions (LUTD) in patients who failed conservative therapies, such as behavioral and pharmacological strategies. SNM mechanism of action relates to the effects of electrical stimulation on afferent and efferent nerve fibers connecting the bladder and pelvic floor to spinal interneurons and central nerve system (CNS) ( 1 ). Since SNM influences sacral afferents and modulates spinal cord reflexes and brain centers which control the lower urinary tract (LUT), this therapy is usually indicated for patients whose neural system is intact or is partially damaged. Most studies on SNM focused on the role of this minimally invasive treatment in patients presenting idiopathic overactive bladder (iOAB), chronic non-obstructive urinary retention and chronic pelvic pain. However, there is increasing evidence to suggest that SNM may also be effective and safe in selected neurological patients presenting LUTD ( 1 - 3 ).

According to the International Continence Society (ICS), adult neurogenic lower urinary tract dysfunction (ANLUTD) refers to ‘abnormal or difficult function of the bladder, urethra (and/or prostate in men) in mature individuals in the context of clinically confirmed relevant neurologic disorder’ ( 4 ). Neurogenic Overactive Bladder (nOAB) is characterized by ‘urgency, with or without urgency urinary incontinence, usually with increased daytime frequency and nocturia in the setting of a clinically relevant neurologic disorder with at least partially preserved sensation’ ( 4 ). Neurogenic OAB is a common presentation of several neurologic diseases, including CNS lesions (stroke, Parkinson’s disease, tumors, etc.) and spinal cord lesions. Studies on SNM for patients with neurological diseases tend to follow the same criteria used for patients with idiopathic LUTD. Indications have included refractory neurogenic detrusor overactivity (DO), urinary retention (UR) due to detrusor underactivity (DU) or detrusor sphincter dyssynergia (DSD), and fecal incontinence (FI) ( 1 ). This article aims to review the available evidence on SNM for patients with distinct neurological diseases, highlighting current barriers and latest technological developments, which may impact the urological practice.

## MATERIALS AND METHODS

A PubMed database search was conducted in April 2020 using the following Medical Subject Heading (MeSH) terms: ‘sacral neuromodulation’ and either ‘neurogenic’ or ‘spinal cord’ or ‘multiple sclerosis’ or ‘Parkinson’ or ‘cerebrovascular accident’ or ‘spinal bifida’ or ‘disk surgery’. Multiple free text searches were performed using the following terms individually through all fields of the records: ‘neuromodulation’, ‘neurogenic’, and ‘neurogenic bladder’. The 2017-2019 abstract volumes of the American Urological Association (AUA), Society of Urodynamics, Female Pelvic Medicine & Urogenital Reconstruction (SUFU), European Association of Urology (EAU) and International Continence Society (ICS) were also retrieved and reviewed. The search was restricted to the English language.

### Available evidence on NLUTD

SNM for patients with neurological diseases is increasing, although it is an ‘off label’ indication and the Federal Drug Administration (FDA) has not yet approved SNM for this specific population. Most case series on SNM for neurogenic LUTD used staged procedures and test phase success was defined as ≥50% symptom improvement in bladder and/or bowel diaries ( 1 ). According to available evidence, most neurological patients who underwent SNM were at low risk for upper urinary tract (UUT) deterioration. Reports across the literature lacked standardized criteria for patient selection, success definition, follow-up, which precluded performing comparations and meta-analysis. SNM has been used in different neurological diseases, such as incomplete sacral cord injury (SCI), multiple sclerosis (MS), Parkinson´s disease (PD), cerebrovascular accident (CVA), cerebral palsy (CP), and brain trauma (Level of Evidence: III, Grade of Recommendation: C) ( 5 ).

### SNM in Spinal Cord Injured (SCI) patients

SNM has been used mostly in patients with incomplete spinal cord lesions. A recent meta-analysis shows success rates of 46% and 76% for the test and permanent phases, respectively ( 6 ). Adverse events included change in stimulation sensation, loss of efficacy, pain or spasticity in the lower limbs, pain at the implantable pulse generator site, adverse change in bowel function, and wound infection.

Almost all the studies on SCI were case series (LE 4) ( 6 ). ICS best practice statement on SNM recommends that SNM should be limited to ASIA D and E in patients with preserved bladder filling sensation ( 5 ). Success rate of SNM in patients with upper motor neuron injury may be higher than in patients with lower motor neuron injury, since the former preserves afferent integrity and contractility of the detrusor ( 7 ).

### SNM in Multiple Sclerosis (MS) patients

SNM has shown promising results in MS patients who presented with DO and DSD, but a low success rate has been reported for those patients with UR due to neurogenic detrusor underactivity ( 8 ). According to a review by Puccini et al. SNM test phase success rate was around 60%, with a final subjective cure rate of 45% and a global satisfaction of 85% in patients with MS ( 9 ). Median Expanded Disability Status Scale (EDSS) at baseline was 5 in most studies. Complications included primary failure (25%), perceived pain and discomfort at the site of the IPG (25 to 56%, and 40%, respectively), lead migration (11 to 20%), need for reoperations (6 to 50%), among others (neuropathic pain, hypersensitivity to stimulation, and infections; 6 to 15%) ( 9 ). Usually, MS patients who are candidates for SNM should have stable disease without an expected requirement for frequent or routine magnetic resonance imaging (MRI); patients with rapidly progressive MS typically should not have SNM systems implanted ( 5 ).

### SNM for Parkinson’s Disease (PD)

A recent publication reported 20 PD patients, most of them (88%) presenting neurogenic DO and failure to antimuscarinics (median age 74years). Test phase with percutaneous needle evaluation (PNE) was done in 6 patients (30%), and the remaining 14 (70%) underwent staged procedure with tined-lead electrodes ( 10 ). Thirteen patients (65%) presented clinically relevant response (>50% improvement) after a median test phase duration of 8.7 days and received IPG implant. After a median follow-up of 20 months, only seven patients reported continuous storage LUTS improvement (>50% compared to baseline; intent-to-treat efficacy: 35%), which favored staged procedures with tined-lead electrodes (PNE: 0% vs. Tined-Lead: 50% improvement; p=0.05). Four device explantations were performed (three due to loss of efficacy, and one due to discomfort) ( 10 ).

### SNM for spina bifida

The largest series on SNM for spinal bifida reported poor results and high rates of complications ( 11 ). Indications were FI or UI, constipation, UR, or a combination of these conditions. Median number episodes of FI during the test phase with PNE decreased significantly from 8.5 to 3.5. Only 3/10 (30%) patients had more than 50% improvement and proceed to the tined-lead and IPG implant, which resulted in a good final response in 2 patients.

### SNM for other neurological diseases

Several series had reported outcomes of SNM in patients with various neurological diseases and LUT dysfunction. Okafor et al. reported a case series of 80 patients in which neurologic diagnoses were SCI in 28.8%, MS in 23.7%, stroke in 15%, cerebral palsy in 12.5%, peripheral nervous system disorders in 12.5%, and PD in 7.5% ( 12 ). Urgency urinary incontinence was the primary indication for SNM in 50 (62.5%). Progression to stage 2 SNM was 90%. Revision rates were 46%, with an explantation rate of 33% (most common reason was loss of efficacy). The authors concluded that specific neurologic diagnosis was not predictive of SNM success, revision, or explantation rates.

Kessler et al. performed a systematic review on SNM for NLUTD (studies published up to 2010) and reported pooled success rate of 68% for the test phase and of 92% for permanent SNM, as well as a pooled adverse event rate of 0% or the test phase and of 24% for permanent SNM. Stratifying the outcomes by neurological disease, test phase was successful in 42% for CVA, 100% for CP, 60% for disk disease, and 83% for pelvic surgery. Successful outcomes until the last follow-up after permanent neuromodulation implant was 60% for CVA, 100% CP, 61% pelvic surgery and 56% for disk disease ( 2 ).

Main outcomes of recently published studies (2011-2019) ( 8 , 10 - 12 , 13 - 24 ) on SNM for NLUTD are presented in Table-1 .

**Table 1:** Studies on SNM for NLUTD (published from 2011 to 2019).

Reference and level of evidence (LE)	Year Study type	Underlying neurological disease (indication for SNM)	N	Definition of Success during test phase	Outcomes
Arlen et al. ( 13 ) LE 4	2011 RCS Case-control study	Patients with or without prior spinal surgery (OAB, NOR)	32	≥ 50% symptom improvement in bladder diaries	Clinical success achieved in selected patients with LUTS and a history of spinal surgery; urge incontinence less likely to improve. Mean follow-up of 2.3 years; Complications not reported
Lombardi et al. ( 14 ) LE 4	2011 PCS	Incomplete spinal cord lesions (OAB and/or NOR)	75	≥ 50% symptom improvement in bladder and bowel diaries	14/37 (38%) subjects with two functional pelvic dysfunctions maintained notable clinical improvement with a median follow-up >3 years
Chaabane et al. ( 15 ) LE 4	2011 RCS	Various neurological diseases* (OAB in 34, NOR in 15, NOR+DO in 13)	62*	Clinical and urodynamic improvement ≥ 50% and symptom recurrence after stopping stimulation	41 (66.1%) had more than 50% improvement on urodynamic evaluation and bladder diary and 37 were implanted. With a mean follow-up of 4.3 ± 3.7 years, results were maintained in 28 (75.7%). SNM failed on average 12.0 ± 12.4 months after implantation
Minardi et al. ( 8 ) LE 4	2012 RCS	MS (OAB, NOR)	25	> 50% symptom improvement in bladder diaries and/or >50% decrease in daily catheterizations and increase in voided volumes	15 (60%) patients received the IPG. After a mean follow-up of 61.2 months, 10 patients still had a functioning device. SNM did not help MS patients with urinary retention due to detrusor underactivity.
Lansen-Koch et al. ( 11 ) LE 4	2012 RCS	Spina bifida (Fecal or urinary incontinence, constipation, NOR or a combination)	10	≥ 50% symptom improvement	Only 3/10 (30%) patients succeeded and received the permanent IPG. In one patient the electrode could not be implanted; .one patient developed skin erosion at the stimulator site in the buttock, requiring replacement to the abdomen.
Groen et al. ( 16 ) LE 4	2012 RCS	Spina bifida (Unclear pattern of NLUTD; 2 patients with UI)	3	≥ 50% symptom improvement	The IPG was removed in all 3 patients due to disappointing results (time from implantation to removal was not reported).
Peters et al. ( 3 ) LE 4	2013 PCS	NLUTD (Stroke in 17, MS in 13, PD in 10, incomplete SCI in 4, and others)	71	≥ 50% symptom improvement in bladder diaries	63 of 71 (88.7%) with a neurological disease and 241 of 269 (89.6%) without a neurological disease received the IPG (P = .82). Complications, revisions/explants, and reprogramming sessions were similar in the 2 groups
Lombardi et al. ( 17 ) LE 4	2013 RCS	Incomplete SCI (ASIA C or D) (NOR)	77	50% reduction of volume per catheterization and number of catheterizations per day	11/29 patients (31%) reached a BCI > 100. Most voided with Valsalva maneuver, with vesical pressure 72-95cm H20. 10/29 patients became nonresponsive in a mean follow-up of 54 months.
Andretta et al. ( 18 ) LE 4	2014 RCS	MS (Storage in 41%, voiding in 24%, mixed in 35%)	17	Not stated	75% had significant and lasting improvement in LUTS and in quality of life. SNM was discontinued after a mean time of 66 months due to disease progression in 2 cases and loss of efficacy in 3.
Lombardi et al. ( 19 ) LE 2b	2014 PCS	Incomplete SCI (NOR)	50	Concomitant reduction by at least 50% of volume per catheterization and catheterizations per day	36 patients received the IPG. Significant increase in urinary flow and decrease in residual urine were documented. 11/34 patients at follow-up were ‘inconstant responders’, as they returned to baseline symptoms but responded again with an implant on the contralateral S3. All but one failure occurred more than 3 years after the previous implant.
Chen et al. ( 20 ) LE 4	2015 RCS	Incomplete SCI (Neurogenic bladder and bowel dysfunction)	23	At least 50% clinical improvement (bladder diary, residual volume and the Wexner questionnaire for constipation)	IPG implanted in 13 (56.5%) patients, including 4 who still used intermittent catheterization. During a mean follow-up of 17.5 months, 1 patient failed and 1 patient developed bilateral vesicoureteral reflux.
Engeler et al. ( 21 ) LE 4	2015 PCS	MS (OAB, or LUTS caused by detrusor underactivity or detrusor sphincter dyssynergia, or both)	17	>70 % improvement in voiding and storage symptoms on voiding diary	At 3 years of follow-up, voided volume improved from 125 to 265 mL, post void residual from 170 to 25 mL, micturition frequency from 12 to 7/day and number of UI episodes/day from 3 to 0. Satisfaction was 80%. Loss of clinical benefit in 2 patients; there were no major complications.
Wöllner et al. ( 22 ) LE 4	2016 RCS	Various neurological diseases Incomplete SCI in 35 patients (70%) (Neurogenic DO, neurogenic urinary retention)	50	Objective improvement of voiding frequency and daily pad usage, or post-void residual urine.	IPG implanted in 35 patients (70%). In 26 patients with refractory DO, daily frequency was reduced from 9 to 6, and pad use was improved from 2.6 to 0.6/day; nine patients with NOR had post void residue reduced from 370 to 59 mL; At the last follow-up, SNM was in use in 32 (64%) patients.
Okafor et al. ( 12 ) LE 4	2016 RCS	Various neurological diseases	80**	Not stated	Progression to stage 2 SNM was 90%. Revision rate=46%; Explantation rate=33% (most common reason was loss of efficacy).
Greenberg et al. ( 23 ) LE 4	2019 RCS	Parkinson Disease	14	≥ 50% symptomatic improvement	IPG implanted in 8 patients. Decreased urinary frequency up to 18 months compared to baseline: 7.70±8 voids/24 hours vs. 15.6±2.2 voids/24 hours (p<0.05). No patients required explantation of their SNM device.
Sharifiaghdas ( 24 ) LE 4	2019 RCS	Spinal dysraphism in 6 and traumatic spinal cord in 2	8	≥ 50% reduction of UI, in urinary frequency, post-void residual volume and need for intermittent catheterization	Positive clinical response was achieved in seven (85%) at a mean follow-up of 14.25 months. Three patients became capable to stop clean intermittent catheterization
Peyronnet et al.( 10 ) LE 4	2019 RCS	Parkinson´s Disease	20	≥ 50% reduction in storage symptoms	IPG implanted in 13 patients, 7 patients still presented response at 20-month follow-up. Four explanations of the device were performed due to loss of efficacy (n= 3) and ‘discomfort’ (n=1).

**RCS** = retrospective case series; **P** = permanent sacral neuromodulation; **T** = test phase; **PCS** = prospective cohort study; **NR** = not reported; **CR** = case report; **LE** = level of evidence; **OAB** = overactive bladder syndrome; **NON** = non-obstructive retention; **DO** = detrusor overactivity; **DSD** = detrusor-sphincter dyssynergia; **SNM** = sacral neuromodulation; **LUTS** = lower urinary tract symptoms; **NLUTD** = neurogenic lower urinary tract dysfunction; **BCI** = bladder contractility index, **MS** = multiple sclerosis, **PD** = Parkinson’s disease, **SCI** = spinal cord injury, **IPG** = implantable pulse generator; **UI** = urinary incontinence.

* n = 62 patients: Multiple sclerosis = 13; Incomplete spinal cord injury = 13; Peripheral neuropathy = 8; Parkinson’s disease = 4; Myelitis/encephalitis = 4; Stroke = 4; Acquired brain injuries = 3; Cerebral palsy = 2; Central nervous system tumor = 2; other = 9.

**n= 80 patient: SCI = 23; MS = 19; Stroke = 12; cerebral palsy = 10; peripheral nervous system disorders = 10; PD = 6.

### Main barriers and latest developments

Some of the characteristics of the current SNM devices may limit their use in the urological practice, especially for neuro-urological patients.

### Size

Although the IPG is generally well tolerated in the gluteal region, thin patients may feel the device under the skin and report discomfort when lying down, which does not improve when the stimulation is turned off. In selected cases a reoperation might be needed, in order to release the pseudocapsule surrounding the generator ( 25 ). This unusual complication can be avoided by the use of smaller generators that are better suited to the subcutaneous tissue.

**Figure 1:**
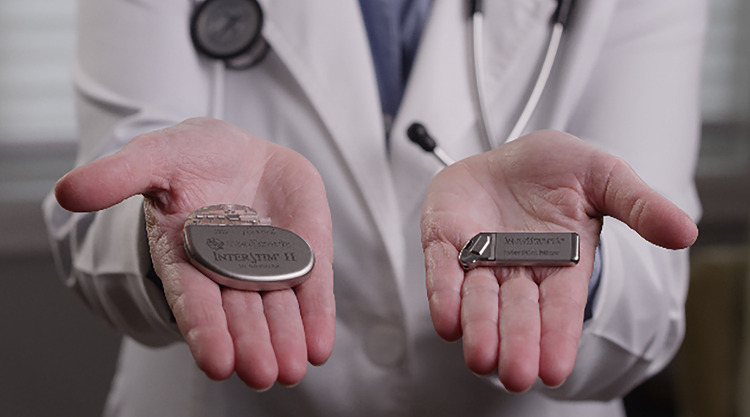
Size difference between the InterStim™ II (non-rechargeable) and the InterStim™ Micro (rechargeable) implantable pulse generators.

**Figure 2:**
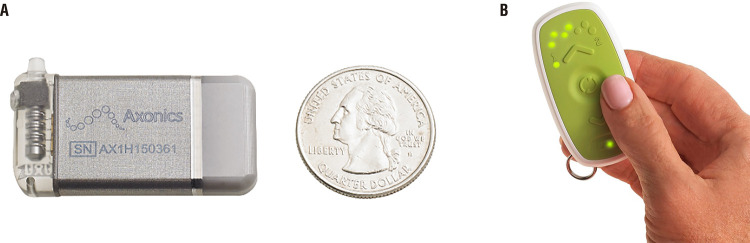
Axonics r-SNM™ implantable pulse generator (A) and patient therapy controller (B).

Newer devices have a significantly reduced size, which facilitates implantation and theoretically increases tolerance to it. Such advance was only possible due to IPG rechargeable capacities. Until recently, Interstim™ II (Medtronic, Minneapolis, MN, USA) was the sole device on the market used to deliver SNM. Actual size of the non-rechargeable InterStim™ II system is 14cm3, while the rechargeable Axonics r-SNM™ (Axonics Modulation Technologies, Inc., Irvine, CA) system is 5.5cm3. The newer InterStim™ Micro technology (2.8cm3) had a volume reduction of about 80% when compared to the standard InterStim™ II system and is approximately 49% smaller in comparison to the Axonics rechargeable SNM device ( 26 ) ( Figures 1 and 2 ).

Nevertheless, smaller implantable generators may also impose specific barriers to obese patients. As the distance and angle between IPG and recharger may change over time due to weigh gain, recharging sessions might be hindered. Twiddler’s syndrome is another potential complication. First described in 1968, pacemaker twiddler’s syndrome refers to permanent malfunction of an implantable device due to patient’s manipulation of the pulse generator. The sequence of symptoms begins with the patient’s deliberate or subconscious spinning of the pacemaker’s pulse generator in a capacious pocket, which may result in subsequent dislodging of the leads ( 27 ).

### Infection

Fortunately, pocket infection is not a frequent complication (3-10%) ( 28 ). Risk factors have been proposed, such as comorbidities, longer duration of test phase (stage 1 ), need for surgical reinterventions and pocket hematoma. So far, there is no clear evidence whether a history of neurological disease would be a risk factor for infection ( 29 ).

For high-risk cases (e.g. renal insufficiency, diabetes mellitus, systemic anticoagulation with heparin, warfarin or novel oral anticoagulants, chronic corticosteroid use, prior lead or IPG site infection), Kolek et al. proposed protecting the generator by wrapping it in an absorbable mesh bag impregnated with minocycline and rifampicin known as TYRX™ (Medtronic, Minneapolis, MN) already used and tested in cardiac pacemaker implants ( 30 ). However, such strategy has not yet been reported in SNM patients.

### Magnetic resonance imaging (MRI)

MRI in the presence of an implanted electrode nearby a sacral nerve root can theoretically cause lead migration or heating, which may result in painful stimulation, damage to the whole SNM system or to the nerve itself ( 31 ). Guzman-Negron et al. sought to determine the safety of SNM in patients during lumbosacral 1.5 Tesla MRI and found no significant adverse events in 11 patients implanted with a Medtronic InterStim™ II device ( 32 ). Lower back pain, which was noted by 6 patients, was the most common indication for imaging. Immediately after magnetic resonance imaging only 1 patient reported mild discomfort during imaging at the site of the implantable pulse generator. This discomfort was present only during the scan and not afterward. Therapeutic efficacy of sacral neuromodulation was unchanged 1 month after imaging ( 32 ).

In neurological subpopulations, such as MS, the lack of MRI compatibility has been considered a relative contraindication to SNM even though clinical benefits have been demonstrated ( 5 ). It is estimated that half of the patients with neuromodulation devices and pacemakers will need a MRI study over lifetime. Actually, 23% of device explantations are currently done for this reason ( 32 ).

It has been shown that in most cases the use of RNM of 1.5 Tesla in the head area is safe and can be performed ( 33 ). In September 2019, FDA approved the Axonics r-SNM™ System for full-body 1.5 Tesla MRI scans. This differs from the recommendation for the standard InterStim™ II device, which is only approved for 1.5 T head MRI. Medtronic has recently received CE Mark approval for its InterStim™ Micro neurostimulator and InterStim™ SureScan MRI leads in January 2020. By making full-body MRI scans possible, this new technology has increased accessibility to sacral neuromodulation (SNM) therapy for European patients. The SureScan leads, which will be used in both the InterStim™ Micro system and in future implants of the existing recharge-free InterStim™ II, are designed to allow for full-body 1.5 and 3 Tesla MRI-conditional scans. Medtronic claims that it is the only company in Europe to provide patients with a choice between rechargeable and recharge-free systems, which are both full-body MRI-conditional.

### Battery

In current non-rechargeable systems, the need for IPG replacement over time may be seen as a technological limitation. Usually, when the battery runs out, the entire generator must be replaced leading to cost overruns. A new generation of rechargeable IPGs has been developed, which allows a significant extension of the life of the device. The Axonics® Sacral Neuromodulation (r-SNM™) System, inclusive of a rechargeable IPG, is designed, tested, and validated for at least 15 years performance in the body. The r-SNM™ system has initially received regulatory approval in Europe, Canada, and Australia for the treatment of overactive bladder, non-obstructive urinary retention, and fecal incontinence ( 34 ). As mentioned above, Medtronic has also got approval in Europe for its rechargeable system (InterStim™ Micro). Rechargeable systems should be charged every 2 to 3 weeks and allows high-energy to be used ( 34 ).

Although some of the new advantages of the devices that are on the way are very promising, it is still not very clear how to choose the ideal candidate for each of them. Rechargeable devices provide a longer battery life, which can last up to 15 years (compared to 5 to 7 years for non-rechargeable batteries), so they can sometimes exceed the patient’s life expectancy. On the other hand, recharging process requires training and manual skill on the part of the patient, being determining factors for proper functioning. With the use of new standardized SNM implantation techniques, battery depletion rate has been reduced, which could extend its useful life without the need for rechargeable devices ( 35 ). It must be recognized that rechargeable devices impose more frequent interaction between the patient and the equipment, which is avoided with a properly configured non-rechargeable device, as the patient usually forgets about the implanted device and underlying disease. Therefore, the ideal patients for a rechargeable device must be carefully chosen, paying attention to factors such as life expectancy, the ability to handle the equipment, the location and size of the required pocket, and the need for high-energy use (mainly in the management of chronic pain and neurogenic LUTD), which can compromise IPG useful life.

## CONCLUSIONS

Available studies on SNM for neurogenic LUTD are based on small sample sizes and heterogeneous populations, which are incompletely characterized in terms of severity of neurologic impairment, lacking standardized definitions of success and follow-up. Newer technologies, such as rechargeable and MRI-compatibility devices, may help overcome well-known barriers to the dissemination of the SNM in neuro-urological patients. Further prospective studies with larger sample sizes, appropriate disease classification, standardized definitions of success, and longer follow-up with special attention to failure and complication rates are still needed to define which subgroups of neurological patients benefit the most from this minimally invasive technique.
